# Physiological, Pathological, and Circadian Factors Impacting Skin Hydration

**DOI:** 10.7759/cureus.27666

**Published:** 2022-08-04

**Authors:** Jose V Camilion, Siya Khanna, Sheela Anasseri, Coral Laney, Harvey N Mayrovitz

**Affiliations:** 1 College of Medicine, Nova Southeastern University Dr. Kiran C. Patel College of Osteopathic Medicine, Fort Lauderdale, USA; 2 Osteopathic Medicine, Nova Southeastern University Dr. Kiran C. Patel College of Osteopathic Medicine, Fort Lauderdale, USA; 3 Medical Education, Nova Southeastern University Dr. Kiran C. Patel College of Allopathic Medicine, Davie, USA

**Keywords:** psoriasis, atopic dermatitis, circadian rhythm, aquaporins, nutritional factors, age effects, skin barrier, stratum corneum, transepidermal water loss, skin water

## Abstract

Thismanuscript focuses on the physiological, environmental, nutritional, circadian, and aging factors affecting skin tissue water and hydration parameters. The literature findings indicate a multiplicity of interacting processes among these parameters, ultimately impacting skin hydration in normal skin and playing a role in conditions such as atopic dermatitis and psoriasis. The maintenance of adequate skin hydration, aided by the proper functioning of the skin’s protective barrier, is facilitated by stratum corneum integrity with the presence of tight junctions and lipids such as ceramides, each of which is impacted by changes in most of the evaluated parameters. Abnormalities in aquaporin 3 (AQP3) expression and associated deficits in skin hydration appear to have a role in atopic dermatitis and psoriasis. AQP3 hydration-related aspects are influenced by circadian rhythms via modulations associated with CLOCK genes that alter AQP3 protein expression. Ultraviolet exposure, aging, and low temperatures are among those factors that affect skin ceramide composition, potentially leading to increased transepidermal water loss and negatively impacting skin hydration. Vitamin C, collagen, and probiotics may increase ceramide production and improve skin hydration. The extent to which each of the different evaluated factors affects skin hydration varies but is usually large enough to consider their potential effects when investigating skin in research and clinical settings.

## Introduction and background

As the largest organ of the body, the skin plays many essential roles, such as protecting the body against pathogens, regulating body temperature, cell fluid maintenance, synthesis of vitamin D, and detection of stimuli [1]. Parameters used to assess the condition of skin related to its hydration status include tissue water determined by measurements of the stratum corneum [2], dermis and deeper skin layers [3] and measures of water loss via transepidermal water loss (TEWL) measures [4]. These parameters are determined and affected by multiple physiological and pathological factors, the analysis of which is the main subject of the present study. Knowledge about the specific factors impacting these skin parameters is important to a deeper understanding of how the skin maintains its proper functions, the cause of abnormalities that may occur, and the effect of correction and care.

## Review

The focus of this report is to review and discuss the physiological, environmental, nutritional, and aging factors that affect skin tissue water and hydration parameters. The goal is to provide a structured and up-to-date, critically examined compilation to help enhance understanding of normal and pathological variations in skin properties. 

Physiological processes involved 

Skin hydration parameters are dependent on multiple structural and physiological processes, including the composition of the skin barrier, the cell types and features involved, and intrinsic and extrinsic modulating factors. In this section, these will be sequentially reviewed. 

Composition of the Skin Barrier

The integumentary system is made of the epidermis, dermis, hypodermis, associated glands, hair, and nails [1]. The epidermis is the body’s first line of defense and works to form an effective barrier between the internal and external environments of the organism [5,6]. The three main functions of the skin barrier are to limit passive water loss, restrict absorption of chemicals, and to prevent infections [5]. Epidermal layers are formed by the process of keratinization, in which keratinocytes proliferate and mature to different stages. This leads to the formation of the stratum basale (innermost layer), stratum spinosum, stratum granulosum, and the outermost layer, the stratum corneum (SC) [5]. The SC is essential for maintaining an effective epidermal barrier against excess loss of water, ions, and proteins [5]. Tight junctions are transmembrane proteins that control how water and solutes are communicated between cells [7]. A high concentration of tight junctions is found where the stratum granulosum meets the SC, which makes these structures significant in transepidermal water absorption and water loss. Pathologies that target the expression or integrity of epidermal tight junctions will, therefore, affect skin permeability [7]. Skin hydration is frequently measured by assessing the electrical capacitance of the SC since the capacitance is directly proportional to the moisture content and hydration presented in arbitrary units [8]. Numerical data for SC hydration in the present report is thus always expressed without units. Sometimes hydration includes the measurement of the dermis as well, and this is assessed by measuring the tissue dielectric constant that is also directly related to the water content [9-13].

Role of Corneocytes and Its Components in Skin Hydration

Corneocytes are largely made up of the protein keratin and are nonliving cells devoid of a nucleus. These cells make up most of the SC and help maintain transepidermal water loss (TEWL) at normal values. They are arranged in a “brick and mortar” pattern and play a critical role in forming the skin barrier [5]. Components of corneocytes importantly include natural moisturizing factor (NMF) derived from the proteolytic breakdown of the filaggrin protein [14]. NMF, together with other corneocyte components, have water binding and retaining properties allowing corneocytes to absorb three times their weight in water. A deficiency in any of these components can lead to dehydrated skin, excessive water loss, and pathologic conditions [15]. The conversion of filaggrin to NMF depends on the hydration status of the skin, and dysregulation of this process or reduced levels of NMF may be associated with several skin conditions, including atopic dermatitis, ichthyosis, xerosis, and psoriasis [14]. 

The NMF of skin is mainly composed of free amino acids, pyrrolidine carboxylic acid, urocanic acid, lactic acid, and urea [16]. Of the free amino acids, the most abundant are L-serine (~36%), glycine (~22%), and L-alanine (~13%) [17]. As noted, the main function of NMF is to help maintain proper skin hydration by drawing and binding water from the atmosphere, given the highly water soluble and hygroscopic nature of its constituents [14,18]. Reductions in the levels of NMF have been associated with dry skin, scaling, flaking, fissuring, and crackling [14]. In addition to NMF, there are other hygroscopic agents, such as glycerol, within corneocytes that absorb moisture from the air and assist in forming the barrier to TEWL [19]. Another important component in the regulation of skin hydration is hyaluronic acid, a non-protein-glycosaminoglycan that helps to maintain the structural integrity and barrier function of the skin because of its water-binding properties [15]. Hyaluronic acid also interacts with keratinocytes to regulate lipid synthesis and keratinocyte differentiation [15]. The intercellular lipids in the SC form a series of parallel lamellar membranes and play an important role in creating and maintaining an effective skin barrier function to maintain TEWL at appropriate levels. The SC has multiple types of lipids, including ceramides, free fatty acids, and cholesterol. These three major lipids normally are present within very specific ratios to one another, and alterations of the ratios can lead to deterioration of the physiological barrier resulting in an increased TEWL. The decrease of ceramide content, leading to altered lipid ratios, can be caused by increases in pH or inflammation [20,21].

*Role of Aquaporins in Skin Hydration* 

Aquaporins are protein channels that allow the passage of water through cellular membranes [22]. Aquaporin 3 (AQP3) is the most abundant skin aquaporin that facilitates water and glycerin transport into SC to help keep it hydrated [23]. AQP3 is an aquaglyceroporin that is highly expressed in epidermal layers below the SC but with low expression in the SC itself. This arrangement helps the SC to insulate itself from the environment while retaining the water provided by the epidermis. Some plant-derived compounds and products, such as Ajuga turkestanica extract, that regulate AQP3 expression can be used to increase skin hydration [22]. Additionally, AQP3 has been shown to regulate keratinocyte proliferation, cell migration, and tumor genesis [24]. The association between increased AQP3 expression and increased keratinocyte proliferation may result in increased skin hydration. Abnormalities of AQP3 expression are associated with the development of skin conditions such as psoriasis and atopic dermatitis [23-25].

Environmental, nutritional and aging factors affecting skin hydration

Skin hydration and the skin properties that change with altered hydration are affected by environmental, nutritional, and age-related factors. These aspects are considered sequentially in this section. 

*Environmental Factors Affecting Skin Hydration* 

The main environmental factors contributing to decreased skin hydration are UVB exposure, low temperatures, and low humidity. Overexposure to UVB is associated with decreased skin hydration and increased TEWL [26]. The increased TEWL is mainly attributable to the degradation of the skin’s barrier function. Effects of UV on the skin barrier include loss of cell cohesion and mechanical integrity on the SC’s intracellular components, such as intracellular lipids and corneodesmosomes [27].

The seasonal climate also impacts skin hydration. A study that sought to understand the influence of different seasons on SC lipid composition reported that SC lipid levels decreased in winter vs. spring and summer [28]. The study’s outcome indicated a 20% reduction in ceramide 1 lineolate lipid levels assessed in leg skin. These authors also suggested that such reductions in lipids impact skin hydration by increasing the susceptibility of the skin to xerosis and disruptions in barrier function, particularly during winter months, and that barrier resistance can be improved using linoleic acid esters [27]. Furthermore, hot environments were shown to cause the production of more sweat, increasing hydration levels and TEWL [29]. A different study sought to investigate the number of skin pores present depending on the summer and winter seasons. The results showed that there were more pores in the summer compared to the winter, leading the authors to conclude that TEWL and hydration vary with season and body regions [30].

Humidity has also been shown to play a role in epidermal structure and permeability barrier homeostasis. One study demonstrates that exposure to low humidity increases epidermal DNA synthesis in normal murine epidermis. Low humidity stimulates the DNA response to barrier disruption, leading to epidermal hyperplasia. Furthermore, exposure to a dry environment for 48 hours before barrier disruption results in mast cell hypertrophy, degranulation, and inflammation. These changes in environmental humidity include increased keratinocyte proliferation and markers of inflammation [31]. A different study showed a 31% loss of TEWL in animals maintained in a dry versus humid environment over two weeks. Associated with this change was an increase in the thickness of the epidermis, as well as the dry weight of the stratum corneum in a dry environment [32]. 

*Nutritional Factors Affecting Skin Hydration* 

Adequate nutrition and topical skin care are essential to help maintain moisture and elasticity of the skin [33]. As of yet, research on the effects of diet and skin hydration has been primarily focused on vitamin C, calcium, and polyunsaturated fatty acids. Vitamin C as a nutritional supplement to improve skin barrier function has been extensively studied and has been reported to stimulate ceramide production in keratinocytes by modulating metabolic enzymes to improve epidermal barrier function [34]. Ceramides play an essential role in modulating water retention within the SC and pathologies causing a reduction in ceramide content can cause an excess amount of water loss [35]. A proposed mechanism by which Vitamin C supplementation may increase ceramide production involves three pathways: de novo ceramide synthesis, sphingomyelin hydrolysis via substrate regulation of sphingomyelinases, and hydrolysis of sphingosine-1-phosphate by sphingosine-1-phosphate phosphatase [34]. In one study, human keratinocytes were grown in a medium containing 1.2 mM calcium and Vitamin C (50 μg/ml) over 11 days, and ceramide content was assessed [34]. In this study, Vitamin C significantly increased ceramide content via the action of ceramide metabolic enzymes (p<0.01). In addition, keratinocytes displayed an increased activity and expression of the sphingosine-1-phosphate phosphatase enzyme after Vitamin C incubation. Further, Vitamin C, calcium, and linoleic acid aided keratinocyte proliferation and differentiation into the epidermis. These authors suggested that Vitamin C could be used in a clinical setting to enhance barrier function and hydration in disorders involving reduced epidermal ceramide content, such as occurs in atopic dermatitis and psoriasis. 

Calcium is a nutrient also responsible for the maintenance of the epidermal barrier due to its crucial role in regular keratinocyte differentiation. There are multiple pathways, both genomic and non-genomic, involved in the differentiation of keratinocytes [36]. The differentiation of keratinocytes leads to the formation of the different layers of skin, including the SC, which is responsible for the barrier that protects against water loss [5]. A study demonstrated the knockout of calcium-sensing receptor (CaR) in mice, which led to a disturbance of the Ca2+ gradient and impaired keratinocyte differentiation and permeability barrier homeostasis [37]. A different study measured how the elevation of extracellular calcium caused stratification and keratinization of keratinocytes. The earliest changes associated with elevation of calcium levels were the formation of desmosomes between adjacent keratinocytes, promoting adhesion [38]. This study thus demonstrates calcium as a useful manner of determining the adhesions responsible for preventing water loss. 

Polyunsaturated fatty acids found in vegetable oils and fish oils have been found to contain cutaneous anti-inflammatory and antiproliferative metabolites, which may be used in inflammatory skin disorders. Deficiency in linoleic acid, a polyunsaturated acid, has been shown to cause scaly skin disorders and excessive water loss [39]. A study demonstrated that supplementation of gamma-linolenic acid-rich seed oil of borage bypasses a step of hepatic 6-desaturation of fatty acids and may compensate for the lack of essential fatty acids in individuals deficient in delta 6-desaturase. Twenty-nine healthy elderly individuals with a mean age of 68.8 years received a daily dose of 360 or 720 mg GLA for two months using borage oil in gelatine capsules. The consumption of borage oil induced an improvement of cutaneous barrier function in elderly individuals, with a mean decrease of 10.8% in TEWL [40]. A different study performed a randomized double-blind analysis in which healthy adults were given primrose oil (a gamma-linolenic acid-containing vegetable oil) over 12 weeks. Skin moisture, TEWL, elasticity, firmness, fatigue resistance, and roughness were measured and significantly improved by 12.9, 7.7, 4.7, 16.7, 14.2, and 21.7%, respectively [41]. Similar findings on skin hydration and TEWL have been found with flaxseed oil, and safflower seed oil, other polyunsaturated fatty acids [42].

Dietary patterns have also been studied to further understand the role of glycation on skin hydration. In a cross-sectional study of 84 healthy participants between the ages of 19 to 37 years, dietary intake was assessed using a dietary questionnaire over the previous 12 months [43]. Results indicated that a high intake of cereals, starch, potatoes, saccharides, fish, and shellfish was associated with a significant reduction in skin hydration of the forehead (p<0.05) [43]. The association between the diet of skin hydration has yet to be effectively explored and is not yet clear. More studies aimed at the influence of diet and skin hydration should be investigated. 

Collagen Supplementation as a Factor Impacting Skin Hydration

The skin contains a network of collagen fibers that provide structural support and helps maintain the skin’s elastic properties, with collagen peptides having the ability to increase the amount of collagen and water-binding glycosaminoglycans [44]. During an eight-week study, 39 subjects at least 65 years of age were randomized to a treatment or control group [45]. The treatment group consumed an oral nutritional supplement containing trace minerals and vitamins and 10 g of a collagen peptide daily, and the forearm SC was evaluated at baseline and the end of the study. A significant increase in mean hydration was reported in the treatment group from 43.7 at baseline to 51.7 post-intervention (p<0.001), with no change in the control group. A meta-analysis was performed that investigated the effects of hydrolyzed collagen supplementation on skin aging, where 19 different studies were selected, totaling 1,125 participants aged between 20 and 70 years. The study showed favorable results for hydrolyzed collagen compared to placebo in terms of skin hydration and aging on the skin [46]. The effect of a nutritional drink supplement containing 2.5 g of collagen peptides with vitamin C, zinc, biotin, vitamin E, and acerola fruit extract was reported to improve SC hydration and skin elasticity in 72 healthy women over 35 years old [47]. In this study, participants were divided into treatment and placebo groups. The treatment group consumed the nutritional drink supplement daily over 12 weeks. At the 12-week mark, there was a significant increase in mean skin hydration (28% ± 11.5%, p<0.0001) in the group that received the nutritional drink supplement, compared to a 9.0% ± 6.6% increase in mean skin hydration in the placebo group. It is important to highlight that the nutritional drink contained other peptides aside from collagen, and so there are several confounding elements affecting skin hydration aside from collagen in this case.

Glycation as a Factor Impacting Skin Hydration

Glycation is a non-enzymatic process by which protein and lipids are modified upon exposure to sugars [48]. Glycated keratin in the epidermis is associated with a decrease in SC water levels that contributes to skin dryness [49]. This may be due to excess binding of glucose to skin fibers reducing skin elasticity, thereby decreasing hydration [48]. The negative impact of glycation on skin hydration was seen in the skin of persons with diabetes mellitus attributed to increased advanced glycation end products [50]. That study evaluated 49 persons with diabetes mellitus and reported that those with a fasting plasma glucose >110 mg/dL (n =34) vs. those with glucose <110 mg/dL had a lower skin surface hydration with no observed change in TEWL [49]. 

Probiotics as a Factor Impacting Skin Hydration

Probiotics have been reported to promote skin health by preventing eczema, acne, atopic dermatitis, and allergic inflammation [51]. The role of probiotics in skin hydration is a recent subject of interest. In a randomized study, 110 volunteers aged 41 to 59 years who had dry skin and wrinkles were divided into treatment and placebo groups [52]. The treatment group took 1 x 1010 colony forming units of Lactobacillus Plantarum HY7714 daily for 12 weeks. SC hydration was measured every four weeks on the hand dorsum and face. Compared to baseline, TEWL values at four, eight, and 12 weeks were all significantly reduced in both groups (p<0.001). However, at 12 weeks, individuals in the treatment group (n = 81) had a significant increase in skin water content on the face (p<0.01) and the hands (p<0.05), whereas no significant change was observed in the placebo group [52]. A similar study evaluated the effects of the probiotic Bifidobacterium on skin hydration [53]. In this study, skin hydration was evaluated in 101 healthy young Japanese females for four weeks, and thereafter 81 of them consumed a test beverage daily for four weeks whereas the remaining 20 did not consume the test beverage. The test beverage was fermented milk containing *Bifidobacterium breve* strain Yakult (YIT 12272), *Lactococcus lactis* YIT 2027, *Streptococcus thermophilus* YIT 2021, polydextrose, and galactooligosaccharides. After consuming the test beverage, SC hydration levels increased significantly from a baseline level of 23.2 ± 8.2 to 25.1 ± 9.2 (p=0.029), whereas the control group levels were not significantly changed from their baseline levels. These studies suggest a possible beneficial impact of two strains of probiotic consumption on skin hydration levels. Further research on different strains would help evaluate the influence of probiotics on the hydration of the skin. 

Aging as a Factor Affecting Skin Hydration

Age-related reductions in skin hydration have been reported, but the mechanisms responsible are not completely understood [28,45]. In the SC, water is mostly present in the corneocytes and bound to NMF; however a smaller amount is bound to the polar head groups of lipids inside intercellular lamellas [54]. The lateral organization of lipids in the SC allow for its function as a permeable barrier and enable water retention. A previously cited report found all major lipid species, especially ceramides, to be significantly decreased in the SC with increasing age, suggesting a decreased skin barrier function [28]. An age-related reduction in epidermal hyaluronic acid also plays a role in this decrease in moisture due to the relative loss of its water-binding capacity and subsequent skin moisture reduction [55]. Because of its importance, hyaluronic acid has been a target of pharmacological intervention in anti-aging skin treatments that are reported to promote fibroblast activation and skin hydration [56]. Currently, hyaluronic acid itself is used in most procedures aimed at skin rejuvenation [57]. 

Normal circadian, diurnal and ultradian variations 

The circadian rhythm refers to the 24-hour endogenous clock of the body that influences physiologic, metabolic, and behavioral rhythms [58]. Diurnal variation may be defined as a biological rhythm that is synchronized in a day/night cycle. Ultradian rhythms are biological rhythms with a shorter period and higher frequency than circadian rhythms [59]. Circadian rhythms depend on a central regulator located in the suprachiasmatic nucleus of the anterior hypothalamus, with the clock’s function determined by different transcription and translation feedback loops of various circadian clock genes and proteins [60]. The skin itself is made up of numerous cell types and undergoes quantitative changes throughout the day based on intrinsic biological rhythms [61]. These skin biological rhythms cause physiological variations that are influenced by circadian genes. Two circadian genes, one known as Circadian Locomotor Output Cycles Kaput (CLOCK) and the other known as period1 gene (Per1) are expressed in keratinocytes, melanocytes, and dermal fibroblasts and have roles in circadian function within the suprachiasmatic nucleus [62]. CLOCK genes are involved in a mechanism of interlocked transcription-translation feedback loops. When period1 genes reach a certain concentration, CLOCK genes are attenuated, generating 24-hour rhythms [63]. CLOCK genes influence AQP3genes, indicating that CLOCK genes are involved in time-dependent skin hydration [63]. 

Several of the skin’s properties are reported to be subject to circadian variations. These include daily variations in TEWL, keratinocyte proliferation, skin blood flow, and skin temperature [64]. A change related to skin hydration is a higher skin permeability in the evening [65]. Another hydration-related change depends on a circadian variation in AQP3 that affects TEWL [63]. Consequently, TEWL is reported to increase with increased expressions of AQP3 [23].

To date, studies of diurnal influences on the skin have focused on differences in skin properties in the morning vs. evening. Such studies have reported differences in their skin temperature, sebum production, pH, skin hydration, and TEWL, which varied throughout the day [66]. Specific variations in TEWL and stratum corneum hydration have been reported in a study of 16 healthy volunteers who were measured every two hours in two sessions for 24 hours [65]. Time-dependent rhythms were detected in TEWL on the forearm, forehead, upper back, and shin, with TEWL, increased significantly in the evening at all sites, being least in the morning [65]. Additional evidence of TEWL diurnal variations has been reported as well [67,68].

Impacts of clinical conditions on skin hydration 

Several dermatological conditions have skin hydration as an aspect of their pathophysiology, including atopic dermatitis and psoriasis. 

Atopic Dermatitis and Skin Hydration Aspects

Atopic dermatitis (AD) is a chronic and relapsing skin disease characterized by pruritus, inflammation, and skin barrier dysfunction resulting in increased TEWL [5,69]. The exact etiology of AD is unclear, but epidermal barrier dysfunction and immune system dysregulation contribute [5]. Factors contributing to the skin barrier abnormalities include decreased levels of filaggrin protein, lipids, antimicrobial peptides, and disordered tight junctions between cells, as well as genetic and familial factors. Filaggrin gene mutations are thought to be risk factors for developing AD because such mutations lead to cellular disorganization resulting in a “leaky” barrier for water and an increased TEWL [69]. Such an increase in TEWL in AD has been reported to correlate with the severity of AD and be greater in patients with a filaggrin mutation than those without the mutation [4]. 

*Psoriasis and Skin Hydration Aspects* 

Psoriasis is an immune-mediated inflammatory disorder influenced by genes (HLA-Cw6), cytokines (TNF-𝝰), and environmental factors (𝛃-hemolytic streptococcal infections) [70]. Clinical features of psoriasis include skin dryness and erythematous plaques with thick scales with elevated patches with the whitish buildup of dead skin [70]. Sustained inflammation leads to uncontrolled keratinocyte proliferation and dysfunctional differentiation with infiltrates of dermal dendritic cells, macrophages, T-cells, and neutrophils [71]. Psoriasis is associated with increased TEWL and decreased SC water content [72,73]. The reduced stratum water content is reported to be related to the papulosquamous features of psoriasis [74]. A study investigating the role of AQP3 expression in the epidermis found a link between AQP3 expression and skin hydration in psoriasis [23]. In this study, 19 patients with psoriasis were compared with 10 healthy control volunteers for expression of AQP3 via immunofluorescence and by skin hydration measurements on the arm. Compared to control subjects, patients with psoriasis had decreased skin hydration (73.2 ± 10.5 vs. 92.9 ± 10.0, p < 0.001) and increased TEWL (13.5 ± 4.4 vs. 9.75 ± 1.1 g/h/m2, p< 0.002). Other research has implicated AQP3 and phospholipase D2 abnormalities in hyperproliferation in psoriasis and nonmelanoma skin cancers [25]. 

*Circadian Rhythm's Role in Skin Pathology* 

Nocturnal pruritus is a commonly associated symptom of pathological skin states, including psoriasis and AD [64,75]. A possible mechanism underlying this nocturnal pruritus is the increased TEWL in the evening [75], as molecular clocks regulate the expression of APQ3 in the epidermis [60]. The decrease in the epidermal barrier function in the evenings, evidenced by the increased TEWL, could exacerbate the entry of pruritus-inducing agents into the epidermis [75]. In cross-sectional retrospective studies, TEWL has been identified as an effective biomarker and assessment tool to evaluate the intensity of pruritus in patients with AD [76]. To alleviate nocturnal pruritus, there is an increased benefit of using topical treatments at night for AD because of the increased permeability of the skin in AD patients during this time [64,77].

## Conclusions

The focus of this report was to review and discuss the physiological, environmental, nutritional, circadian, and aging factors affecting skin tissue water and hydration parameters. The findings indicate a multiplicity of interacting processes among these parameters, ultimately impacting skin hydration in normal skin and playing a role in conditions such as atopic dermatitis, psoriasis, and others. The skin’s protective barrier that helps prevent water loss via SC components is due to the presence of tight junctions and lipids such as ceramides, each of which is impacted by changes in most of the evaluated parameters. Abnormalities in the expression of AQP3, the most abundant skin aquaporin that facilitates skin hydration, play a role in atopic dermatitis and psoriasis. Circadian rhythms also influence skin hydration via CLOCK genes that alter the expression of AQP3 proteins.

The extent to which each of the different factors affecting skin hydration varies but is usually large enough to consider their potential effects when investigating skin properties in research and clinical settings. Prior UV exposure, aging, and low temperatures affect the lipid composition of ceramides leading to increased TEWL, thereby impacting skin hydration. Additionally, increased glycation as occurs in diabetes mellitus contributes to skin dryness. Conversely, vitamin C, collagen, and probiotics appear to stimulate ceramide production, thereby improving skin hydration. Variations in skin hydration are present in several clinical conditions, including psoriasis, atopic dermatitis, and nocturnal pruritus, and future research into these processes would seem to be warranted. 

## References

[1] Kim JY, Dao H (2021). Physiology, Integument. https://www.ncbi.nlm.nih.gov/books/NBK554386/.

[2] Osseiran S, Cruz JD, Jeong S, Wang H, Fthenakis C, Evans CL (2018). Characterizing stratum corneum structure, barrier function, and chemical content of human skin with coherent Raman scattering imaging. Biomed Opt Express.

[3] Boer M, Duchnik E, Maleszka R, Marchlewicz M (2016). Structural and biophysical characteristics of human skin in maintaining proper epidermal barrier function. Postepy Dermatol Alergol.

[4] Alexander H, Brown S, Danby S, Flohr C (2018). Research techniques made simple: Transepidermal water loss measurement as a research tool. J Invest Dermatol.

[5] Yang G, Seok JK, Kang HC, Cho YY, Lee HS, Lee JY (2020). Skin barrier abnormalities and immune dysfunction in atopic dermatitis. Int J Mol Sci.

[6] Sala-Cunill A, Lazaro M, Herráez L, Quiñones MD, Moro-Moro M, Sanchez I (2018). Basic skin care and topical therapies for atopic dermatitis: Essential approaches and beyond. J Investig Allergol Clin Immunol.

[7] Bäsler K, Bergmann S, Heisig M, Naegel A, Zorn-Kruppa M, Brandner JM (2016). The role of tight junctions in skin barrier function and dermal absorption. J Control Release.

[8] Rim JH, Jo SJ, Park JY, Park BD, Youn JI (2005). Electrical measurement of moisturizing effect on skin hydration and barrier function in psoriasis patients. Clin Exp Dermatol.

[9] Mayrovitz HN, Mikulka A, Woody D (2019). Minimum detectable changes associated with tissue dielectric constant measurements as applicable to assessing lymphedema status. Lymphat Res Biol.

[10] Mayrovitz HN, Arzanova E, Somarriba S, Eisa S (2019). Factors affecting interpretation of tissue dielectric constant (TDC) in assessing breast cancer treatment related lymphedema (BCRL). Lymphology.

[11] Mayrovitz HN, Mahtani SA, Pitts E, Michaelos L (2017). Race-related differences in tissue dielectric constant measured noninvasively at 300 MHz in male and female skin at multiple sites and depths. Skin Res Technol.

[12] Mayrovitz HN, Grammenos A, Corbitt K, Bartos S (2016). Young adult gender differences in forearm skin-to-fat tissue dielectric constant values measured at 300 MHz. Skin Res Technol.

[13] Nuutinen J, Ikäheimo R, Lahtinen T (2004). Validation of a new dielectric device to assess changes of tissue water in skin and subcutaneous fat. Physiol Meas.

[14] Fowler J (2012). Understanding the role of natural moisturizing factor in skin hydration. Practi Dermato.

[15] Verdier-Sévrain S, Bonté F (2007). Skin hydration: a review on its molecular mechanisms. J Cosmet Dermatol.

[16] Maeno K (2019). Direct quantification of natural moisturizing factors in stratum corneum using direct analysis in real time mass spectrometry with inkjet-printing technique. Sci Rep.

[17] Arezki NR, Williams AC, Cobb AJ, Brown MB (2017). Design, synthesis and characterization of linear unnatural amino acids for skin moisturization. Int J Cosmet Sci.

[18] Mojumdar EH, Sparr E (2021). The effect of pH and salt on the molecular structure and dynamics of the skin. Colloids Surf B Biointerfaces.

[19] Rawlings AV, Matts PJ (2005). Stratum corneum moisturization at the molecular level: an update in relation to the dry skin cycle. J Invest Dermatol.

[20] van Smeden J, Bouwstra JA (2016). Stratum corneum lipids: Their role for the skin barrier function in healthy subjects and atopic dermatitis patients. Curr Probl Dermatol.

[21] Levin J, Friedlander SF, Del Rosso JQ (2013). Atopic dermatitis and the stratum corneum: part 2: other structural and functional characteristics of the stratum corneum barrier in atopic skin. J Clin Aesthet Dermatol.

[22] Dumas M, Sadick NS, Noblesse E (2007). Hydrating skin by stimulating biosynthesis of aquaporins. J Drugs Dermatol.

[23] Lee Y, Je YJ, Lee SS (2012). Changes in transepidermal water loss and skin hydration according to expression of aquaporin-3 in psoriasis. Ann Dermatol.

[24] Nakahigashi K, Kabashima K, Ikoma A, Verkman AS, Miyachi Y, Hara-Chikuma M (2011). Upregulation of aquaporin-3 is involved in keratinocyte proliferation and epidermal hyperplasia. J Invest Dermatol.

[25] Voss KE, Bollag RJ, Fussell N, By C, Sheehan DJ, Bollag WB (2011). Abnormal aquaporin-3 protein expression in hyperproliferative skin disorders. Arch Dermatol Res.

[26] Kwon TR, Oh CT, Choi EJ (2016). Conditioned medium from human bone marrow-derived mesenchymal stem cells promotes skin moisturization and effacement of wrinkles in UVB-irradiated SKH-1 hairless mice. Photodermatol Photoimmunol Photomed.

[27] Biniek K, Levi K, Dauskardt RH (2012). Solar UV radiation reduces the barrier function of human skin. Proc Natl Acad Sci U S A.

[28] Rogers J, Harding C, Mayo A, Banks J, Rawlings A (1996). Stratum corneum lipids: the effect of ageing and the seasons. Arch Dermatol Res. Nov.

[29] Kim S, Park JW, Yeon Y, Han JY, Kim E (2019). Influence of exposure to summer environments on skin properties. J Eur Acad Dermatol Venereol.

[30] Song EJ, Lee JA, Park JJ (2015). A study on seasonal variation of skin parameters in Korean males. Int J Cosmet Sci.

[31] Denda M, Sato J, Tsuchiya T, Elias PM, Feingold KR (1998). Low humidity stimulates epidermal DNA synthesis and amplifies the hyperproliferative response to barrier disruption: implication for seasonal exacerbations of inflammatory dermatoses. J Invest Dermatol.

[32] Denda M, Sato J, Masuda Y (1998). Exposure to a dry environment enhances epidermal permeability barrier function. J Invest Dermatol.

[33] Cao C, Xiao Z, Wu Y, Ge C (2020). Diet and skin aging-from the perspective of food nutrition. Nutrients.

[34] Kim KP, Shin KO, Park K, Yun HJ, Mann S, Lee YM, Cho Y (2015). Vitamin C stimulates epidermal ceramide production by regulating its metabolic enzymes. Biomol Ther (Seoul).

[35] Imokawa G, Kuno H, Kawai M (1991). Stratum corneum lipids serve as a bound-water modulator. J Invest Dermatol.

[36] Elsholz F, Harteneck C, Muller W, Friedland K (2014). Calcium--a central regulator of keratinocyte differentiation in health and disease. Eur J Dermatol.

[37] Tu CL, Crumrine DA, Man MQ (2012). Ablation of the calcium-sensing receptor in keratinocytes impairs epidermal differentiation and barrier function. J Invest Dermatol.

[38] Hennings H, Holbrook KA (1983). Calcium regulation of cell-cell contact and differentiation of epidermal cells in culture. An ultrastructural study. Exp Cell Res. Jan.

[39] Ziboh VA, Miller CC, Cho Y (2000). Metabolism of polyunsaturated fatty acids by skin epidermal enzymes: generation of antiinflammatory and antiproliferative metabolites. Am J Clin Nutr.

[40] Brosche T, Platt D (20002000). Effect of borage oil consumption on fatty acid metabolism, transepidermal water loss and skin parameters in elderly people. Arch Gerontol Geriatr.

[41] Muggli R (2005). Systemic evening primrose oil improves the biophysical skin parameters of healthy adults. Int J Cosmet Sci.

[42] Neukam K, De Spirt S, Stahl W, Bejot M, Maurette JM, Tronnier H, Heinrich U (2011). Supplementation of flaxseed oil diminishes skin sensitivity and improves skin barrier function and condition. Skin Pharmacol Physiol.

[43] Lim S, Shin J, Cho Y, Kim KP (2019). Dietary patterns associated with sebum content, skin hydration and pH, and their sex-dependent differences in healthy Korean adults. Nutrients.

[44] Asserin J, Lati E, Shioya T, Prawitt J (2015). The effect of oral collagen peptide supplementation on skin moisture and the dermal collagen network: evidence from an ex vivo model and randomized, placebo-controlled clinical trials. J Cosmet Dermatol.

[45] Nomoto T, Iizaka S (2020). Effect of an oral nutrition supplement containing collagen peptides on stratum corneum hydration and skin elasticity in hospitalized older adults: A multicenter open-label randomized controlled study. Adv Skin Wound Care.

[46] de Miranda RB, Weimer P, Rossi RC (2021). Effects of hydrolyzed collagen supplementation on skin aging: a systematic review and meta-analysis. Int J Dermatol.

[47] Bolke L, Schlippe G, Gerß J, Voss W (2019). A collagen supplement improves skin hydration, elasticity, roughness, and density: Results of a randomized, placebo-controlled, blind study. Nutrients.

[48] Yokota M, Tokudome Y (2016). The effect of glycation on epidermal lipid content, its metabolism and change in barrier function. Skin Pharmacol Physiol.

[49] Sakai S, Kikuchi K, Satoh J, Tagami H, Inoue S (2005). Functional properties of the stratum corneum in patients with diabetes mellitus: similarities to senile xerosis. Br J Dermatol.

[50] de Macedo GM, Nunes S, Barreto T (2016). Skin disorders in diabetes mellitus: an epidemiology and physiopathology review. Diabetol Metab Syndr.

[51] Dolan KE, Pizano JM, Gossard CM (2017). Probiotics and disease: A comprehensive summary—part 6, skin health. Integr Med (Encinitas).

[52] Lee DE, Huh CS, Ra J (2015). Clinical evidence of effects of lactobacillus plantarum HY7714 on skin aging: A randomized, double blind, placebo-controlled study. J Microbiol Biotechnol.

[53] Mori N, Kano M, Masuoka N, Konno T, Suzuki Y, Miyazaki K, Ueki Y (2016). Effect of probiotic and prebiotic fermented milk on skin and intestinal conditions in healthy young female students. Biosci Microbiota Food Health.

[54] Schleusener J, Salazar A, von Hagen J, Lademann J, Darvin ME (2021). Retaining skin barrier function properties of the stratum corneum with components of the natural moisturizing factor-a randomized, placebo-controlled double-blind in vivo study. Molecules.

[55] Papakonstantinou E, Roth M, Karakiulakis G (2012). Hyaluronic acid: A key molecule in skin aging. Dermatoendocrinol.

[56] Göllner I, Voss W, von Hehn U, Kammerer S (2017). Ingestion of an oral hyaluronan solution improves skin hydration, wrinkle reduction, elasticity, and skin roughness: Results of a clinical study. J Evid Based Complementary Altern Med.

[57] Ganceviciene R, Liakou AI, Theodoridis A, Makrantonaki E, Zouboulis CC (2012). Skin anti-aging strategies. Dermatoendocrinol.

[58] Marcheva B, Ramsey KM, Peek CB, Affinati A, Maury E, Bass J (2013). Circadian clocks and metabolism. Handb Exp Pharmacol.

[59] Van Cauter E (1990). Diurnal and ultradian rhythms in human endocrine function: a minireview. Horm Res.

[60] Buhr ED, Takahashi JS (2013). Molecular components of the Mammalian circadian clock. Handb Exp Pharmacol.

[61] Firooz A, Zartab H, Sadr B (2016). Daytime changes of skin biophysical characteristics: A study of hydration, transepidermal water loss, pH, sebum, elasticity, erythema, and color index on middle eastern skin. Indian J Dermatol.

[62] Zanello SB, Jackson DM, Holick MF (2000). Expression of the circadian clock genes clock and period1 in human skin. J Invest Dermatol.

[63] Matsunaga N, Itcho K, Hamamura K (2014). 24-hour rhythm of aquaporin-3 function in the epidermis is regulated by molecular clocks. J Invest Dermatol.

[64] Lyons AB, Moy L, Moy R, Tung R (2019). Circadian rhythm and the skin: A review of the literature. J Clin Aesthet Dermatol.

[65] Yosipovitch G, Xiong GL, Haus E, Sackett-Lundeen L, Ashkenazi I, Maibach HI (1998). Time-dependent variations of the skin barrier function in humans: transepidermal water loss, stratum corneum hydration, skin surface pH, and skin temperature. J Invest Dermatol.

[66] Mayrovitz HN, Berthin T (2021). Assessing potential circadian, diurnal, and ultradian variations in skin biophysical properties. Cureus.

[67] Chilcott RP, Farrar R (2000). Biophysical measurements of human forearm skin in vivo: effects of site, gender, chirality and time. Skin Res Technol.

[68] Reinberg AE, Touitou Y, Soudant E, Bernard D, Bazin R, Mechkouri M (1996). Oral contraceptives alter circadian rhythm parameters of cortisol, melatonin, blood pressure, heart rate, skin blood flow, transepidermal water loss, and skin amino acids of healthy young women. Chronobiol Int.

[69] Nemeth V, Evans J (2021). Eczema. Treasure Island (FL): StatPearls Publishing.

[70] Griffiths CE, Barker JN Pathogenesis and clinical features of psoriasis. Lancet. Jul.

[71] Ayala-Fontánez N, Soler DC, McCormick TS (2016). Current knowledge on psoriasis and autoimmune diseases. Psoriasis (Auckl).

[72] Berardesca E, Maibach HI (1990). Transepidermal water loss and skin surface hydration in the non invasive assessment of stratum corneum function. Derm Beruf Umwelt.

[73] Montero-Vilchez T, Segura-Fernández-Nogueras MV, Pérez-Rodríguez I (2021). Skin barrier function in psoriasis and atopic dermatitis: Transepidermal water loss and temperature as useful tools to assess disease severity. J Clin Med.

[74] Nikam VN, Monteiro RC, Dandakeri S, Bhat RM (2019). Transepidermal water loss in psoriasis: A case-control study. Indian Dermatol Online J.

[75] Patel T, Ishiuji Y, Yosipovitch G (2007). Nocturnal itch: why do we itch at night?. Acta Derm Venereol.

[76] Lee CH, Chuang HY, Shih CC, Jong SB, Chang CH, Yu HS (2006). Transepidermal water loss, serum IgE and beta-endorphin as important and independent biological markers for development of itch intensity in atopic dermatitis. Br J Dermatol.

[77] Duan J, Greenberg EN, Karri SS, Andersen B (2021). The circadian clock and diseases of the skin. FEBS Lett.

